# Genomic Signal Processing Methods for Computation of Alignment-Free Distances from DNA Sequences

**DOI:** 10.1371/journal.pone.0110954

**Published:** 2014-11-13

**Authors:** Ernesto Borrayo, E. Gerardo Mendizabal-Ruiz, Hugo Vélez-Pérez, Rebeca Romo-Vázquez, Adriana P. Mendizabal, J. Alejandro Morales

**Affiliations:** 1 Computer Sciences Department, CUCEI - Universidad de Guadalajara, Guadalajara, México; 2 Molecular Biology Laboratory, Farmacobiology Department, CUCEI - Universidad de Guadalajara, Guadalajara, México; 3 Center for Theoretical Research and High Performance Computing, CUCEI -Universidad de Guadalajara, Guadalajara, México; King Abdullah University of Science and Technology, Saudi Arabia

## Abstract

Genomic signal processing (GSP) refers to the use of digital signal processing (DSP) tools for analyzing genomic data such as DNA sequences. A possible application of GSP that has not been fully explored is the computation of the distance between a pair of sequences. In this work we present GAFD, a novel GSP alignment-free distance computation method. We introduce a DNA sequence-to-signal mapping function based on the employment of doublet values, which increases the number of possible amplitude values for the generated signal. Additionally, we explore the use of three DSP distance metrics as descriptors for categorizing DNA signal fragments. Our results indicate the feasibility of employing GAFD for computing sequence distances and the use of descriptors for characterizing DNA fragments.

## Introduction

Genomic signal processing (GSP) refers to the use of digital signal processing (DSP) tools for analyzing genomic data. Current GSP methods require a step in which a genomic sequence to be analyzed 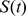 is mapped onto a vector of numerical values (i.e., signal) that represents the information contained in the original sequence [1]. Existing DNA-to-signal mapping methods can be divided into two groups depending on the origin of the numerical values employed. The first group corresponds to methods that assign an arbitrary value to each character *t*, which represents the nucleotides that compose the sequence. Examples of this type of method include 2-bit binary representation (e.g., A = 00, C = 11, G = 10, T = 01) [2], 4-bit binary encoding (e.g., A = 1000, C = 0010, G = 0001, T = 0100) [3], 4-dimensional indicator sequence (Voss representation) (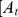 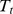 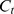 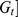 where 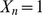 if 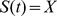) [4], use of a tetrahedron structure [5], use of integer values employing different ranges (e.g., 0 to 3 [6], 1 to 4 [7]), use of real numbers (e.g., A = −1.5, T = 1.5, C = 0.5, G = −0.5 [8], and A = 0.25, G = 0.5, C = 0.75, T = 1 [9]), complex number values (e.g., A = 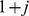, C = 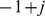, G = 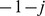 and T = 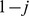 [6], [10]), and use of quaternions (e.g., A = 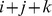, C = 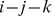, T = 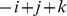, and G = 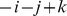 [11]).

The second group includes methods for which the numerical values are defined according to certain biophysical or biochemical properties of the DNA molecules. Examples of this type of mapping include the use of electron-ion interaction potentials (EIIP) (i.e., A = 0.1260, C = 0.1340, T = 0.1335, G = 0.0806) [12] and the use of single atomic numbers (i.e., A = 70, C = 58, T = 66, G = 78) [13]. Other examples include paired nucleotide representations that consider nucleotide complementarity (i.e., A = T = 0, C = G = 1) [14] and graphical approaches such as the DNA-walk model, in which a step is taken upwards (+1) if 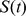 is a pyrimidine (C or T) or downwards (−1) if it is a purine (A or G). Finally, this category also includes Z-curve representation [15], which maps a DNA sequence into a 3-dimensional sequence where 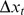 distinguishes between purines and pyrimidines; 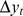 distinguishes between amino-type and keto-type molecules, and 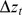 distinguishes between weak and strong hydrogen bonds.

Most GSP methods reported in the literature are focused on the detection of coding regions (e.g., [11], [16]–[23]). In general, these methods consist of performing DNA-to-signal mapping and obtaining the power spectrum of sections of the signal employing the short time discrete Fourier transform (STFT) using a sliding window of fixed length. When a period-3 frequency peak is detected in the power spectra, the section of sequence corresponding to that window is labeled as a coding region. Other applications of GSP include searching for genomic repeats using STFT [24] and determining the structural, thermodynamic, and bending properties of DNA by Fourier analysis [25].

Determining the distance between different genomic sequences is one of the most common types of analysis. In this scope, phylogenetic trees are one of the most essential tools in DNA analysis because they provide structured classification of DNA sequences and enable organization of our growing knowledge of biological diversity. Moreover, this method provides insight into events that occur during evolution. A phylogenetic tree may be constructed from a distance matrix 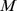 containing a set of values that represents the pairwise distance 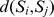 of a set of sequences 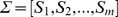. Examples of distance matrix-based methods for phylogenetic tree construction include neighbor-joining [26] and the Fitch-Margoliash method [27], among others.

A distance-matrix corresponding to a set of DNA, RNA, or protein sequences is commonly determined by assessing the distance based on alignment of sequence pairs. Alignment methods have also been used to identify domains, assemble genome contigs, and study sequence variations. Techniques for determining the alignment of a pair of sequences include dot-matrix, dynamic programming, and *k*-tuple methods. In dot matrix-based methods, a recurrence plot is generated by comparing all elements of both sequences to form a two-dimensional matrix in which a dot is placed at the intersection where characters match. Dynamic programming methods compute the optimal alignment between two sequences by considering possible differences due to mutations, insertions, and deletions. This method can also be used for global alignments via the Needleman-Wunsch (NW) algorithm [28] or local alignments via the Smith-Waterman [29] algorithm. The *k*-tuple (word) method attempts to identify sub-sequences of length *k* in the query sequence. Although this method does not guarantee an optimal solution, it is significantly more efficient than dynamic programming, making it suitable for the analysis of large-scale databases. Two of the most popular local alignment methods are FASTA [30] and BLAST [31]. A GSP method has also been proposed for aligning multiple sequences (i.e., MAFFT [32]). In this method, amino acid sequences of different proteins are converted into two numerical vectors consisting of values that correspond to the volume and polarity of the components. The correlation between the two amino acid sequences is computed by the fast Fourier transform (FFT) using a sliding window of fixed length. By assessing the correlation score of both sliding windows, it is possible to detect regions of matching sequences.

Several alignment-free methods for DNA distance computation have been proposed. In general, these methods are based on statistics of word frequencies (i.e., *k*-tuples) using metrics such as weighted Euclidean distance, correlation, co-variance, information theory-based measurements, and angle metrics. [33]. However, other methods based on graphical DNA representations apply dinucleotide (doublet) histograms [34], graph theory [35], trinucleotide (triplet) curves [36], or the average bandwidth of distance/distance (D/D) matrices [37]. A widely used tool for computing phylogenetic trees is the phylogeny inference package (Phylip) [38], which applies different methods such as parsimony, jackknife, bootstrapping, and consensus trees using molecular sequences, gene frequencies, restriction sites and fragments, distance matrices, and discrete characters.

In this paper, we present a novel GSP alignment-free method (GAFD) for determining the distance between two DNA sequences. We introduce a new DNA-to-signal mapping tool that is based on using doublets with a mapping function inspired by *K-strings* [39], which increases the number of possible amplitude values for the generated signal. Additionally, we explore three GSP distance metrics that may be used as descriptors for categorizing dissimilarities between pairs of DNA signal fragments, and that set the basis for developing methods for domain search and characterizing sections of DNA. Our results demonstrate that GAFD performs similarly to the NW and Phylip methods for computing distances among a set of DNA sequences. Moreover, the results obtained using the proposed descriptors show the feasibility of this method in characterizing the types of differences present between sections of sequences. All the methods and algorithms were implemented in MATLAB R2010b. We employed the ARfit module for the autoregressive model computation and signal processing toolbox of MATLAB for FFT computation. NW analysis and phylogenetic tree construction was computed using the bioinformatics toolbox of MATLAB. Source code is available for download at: http://hypatia.cucei.udg.mx/invteorica/DNASignals/.

## Materials and Methods

### DNA sequence-to-signal mapping

Our proposed DNA sequence-to-signal mapping method was inspired by the alignment-free distance method, which employs the nearest-neighbor method (NN) [40], [41]. NN was originally developed for determining the double strand melting temperature [42], [43] and was based on the rationale that the interaction between bases on different strands depends to some extent on the neighboring bases. The model assumes that, under specific environmental conditions, the stability of hydrogen bridges between strands of a nucleic acid duplex for a given doublet and its complementary pairs depends on the identity of its neighboring bases.

Our mapping tool requires that numerical values for all possible combinations of two consecutive bases (doublets) are defined. Let 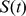 indicate the nucleotide at position 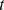 with respect to the beginning of a sequence 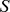 of length 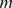. For each doublet, we define a numeric value 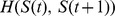. Then, a one-dimensional discrete signal 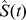 is generated in a manner similar to the *K-strings* approach [39] by combining the values generated by doublets inside a window of magnitude 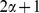:
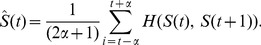
(1)


After mapping the DNA sequence to a discrete signal, a noise reduction method is applied. A typical solution for noise reduction from non-stationary signals is the wavelet denoising method. Wavelet-based noise decomposition of a signal using orthogonal discrete wavelet transform (DWT) can “concentrate” the informative signal into a few wavelet coefficients with large absolute values without modifying the random distribution of noise. Then, DWT-based denoising can be achieved by limiting the number of wavelet coefficients that represent the signal. Consider the model
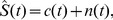
(2)


where 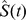 represents the original discrete signal, 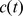 represents the noiseless unknown version of 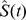, and 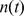 represents the noise. Since DWT is a linear transformation, the wavelet coefficient vectors for each term in Eq. (2) (i.e., 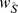, 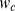, and 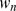) are related by:
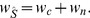
(3)


Denoising is performed by computing the wavelet transform of a signal and then removing the coefficients that correspond to high frequencies by applying a threshold 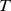. The wavelet coefficients corresponding to low frequencies remain unchanged. The main challenge of this denoising method is determining the 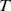 value between small and large wavelet coefficients. To determine this threshold, several algorithms have been proposed recently [44], [45]. In this work, we employed the SureShrink algorithm (i.e., Stein's unbiased risk estimator [46]) because this wavelet denoising method has been successfully applied to biological signals without the loss of important information [47].

### Alignment-free distance computation

Each de-noised signal 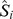 corresponding to a DNA sequence in a set was converted into its frequency representation by applying discrete Fourier transform (DFT) followed by computation of its power spectrum 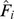. To perform a direct comparison of sequences of different length, we employed zero padding to compute the DFT using the maximum length of all DNA signals in the set. Subsequently, for a given pair of DNA signals, we calculated the alignment-free distance 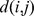 by computing the mean squared error (MSE) of their corresponding power spectrum:
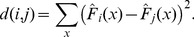
(4)


Finally, a distance matrix 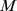 was created by performing pairwise comparison of all sequences in the set.

### Cluster overlapping score

Because the distance matrices generated with GAFD and NW cannot be directly compared, we evaluated these methods by employing a modified version of the cluster overlapping score [48], which is based on the splitting method by Robinson and Foulds. An NJ tree is a dichotomic hierarchic classifier that can be defined by a set of leaves 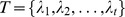 and a set of branches 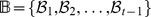. Every branch of the tree is represented in the subset 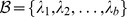, where 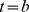 for the root, and 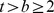 for all other branches of the tree. In this report, a cluster 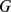 is defined as a branch that is not the root and lies beyond the immediate vicinity of a leaf 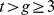. Note that the set of clusters 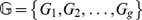 is a subset of 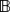.

Given two clusters from two different trees (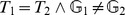) where 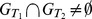, cluster overlapping can be measured using the Dice coefficient as:
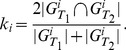
(5)


The cluster overlapping similarity score 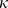 is calculated from the arithmetic mean of all 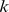 occurrences. Comparison of a tree against itself yields a value of 1.

### GSP distance descriptors

This proposed method for distance computation can determine the global similarity of two DNA sequences. However, this metric may not be capable of determining local differences between the sequences. Therefore, we explored three DSP-based metrics used to generate descriptors that may be useful for characterizing and classifying differences between sections of the sequences.

#### Correlation coefficient

Correlation can be used to measure the dependency of a signal on itself or another signal. For two signals 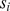 and 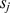 with the same length 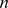, the correlation coefficient is defined as:
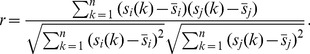
(6)


Depending on the data to be evaluated, the correlation coefficient will be 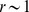 for signals that are highly correlated, 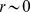 for non-correlated signals, and 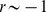 for signals that are inversely correlated. Because we were only concerned with the degree of correlation and not its type, we discarded the sign and defined the descriptor as 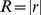.

#### Coherence

Coherence, a relationship measurement used to estimate the degree of linear association between two signals is defined as:
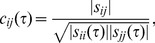
(7)


where 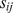 is the cross-spectral density that describes the common power distribution between the two signals, while 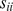 and 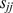 denote the auto-spectral density of 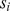 and 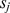 respectively, at a frequency 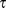. We defined the descriptor 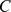 as the mean of the coherences 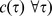. A 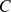 value close to 0 indicates that signals at this frequency are linearly independent, whereas a value close to 1 represents a very high linear correlation. In this work, the spectral densities used for determining the coherence between two signals were computed using an autoregressive (AR) model, which is one of the most widely used tools in DSP [49]. For a given interval, the multidimensional AR model is given by:
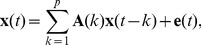
(8)


where 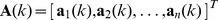 is the 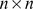 AR coefficients matrix, 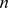 the number of channels, 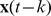 the time-delayed values vector, 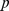 the model order, and 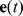 the error vector. To solve Equation 8, it is necessary to fit 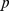 (smaller than the sequence length) and then estimate the AR coefficient matrix [50].

#### Derivative comparison

**Figure 1:**
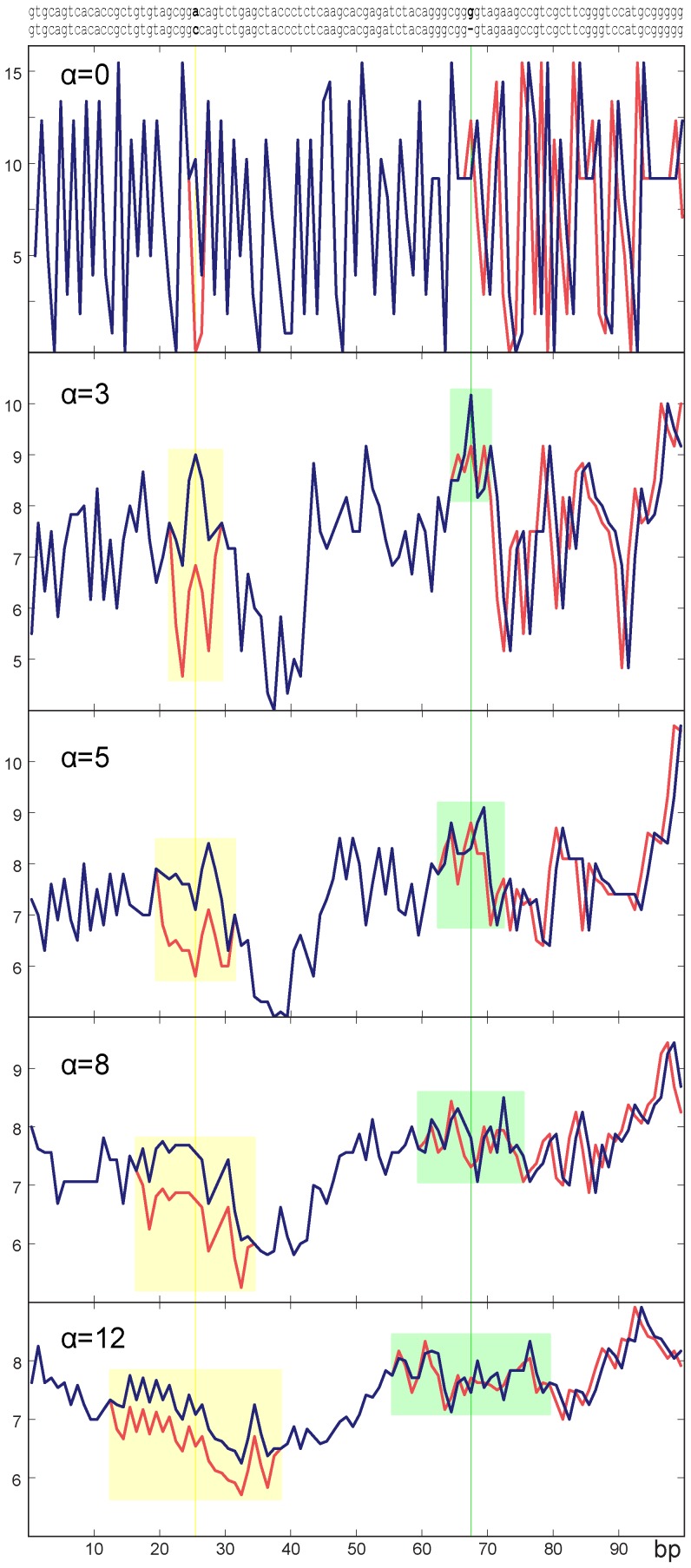
Examples of the resulting DNA sequence-to-signal tool employing different values of *α* and its effects on single sequence changes.

Given that our proposed DNA sequence-to-signal mapping accounts for neighboring nucleotides, small differences between 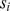 and 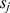 due to indels and mutations produce a shift in the intensity of the resulting signal (Fig. 1). To account for these changes, we compared the derivatives of the two signals by using finite differences and computed the mean slope 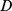 as a descriptor representing the degree of similarity between the two signals. A value 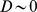 indicates strong similarity.

#### Similarity space

For a given pair of sequences, the three descriptors were used to generate a point with coordinates 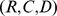 in a three-dimensional space. We hypothesize that it may be possible to characterize sub-sequences by employing clustering or classification methods in this space.

## Results and Discussion

### DNA sequence-to-signal mapping

**Figure 2:**
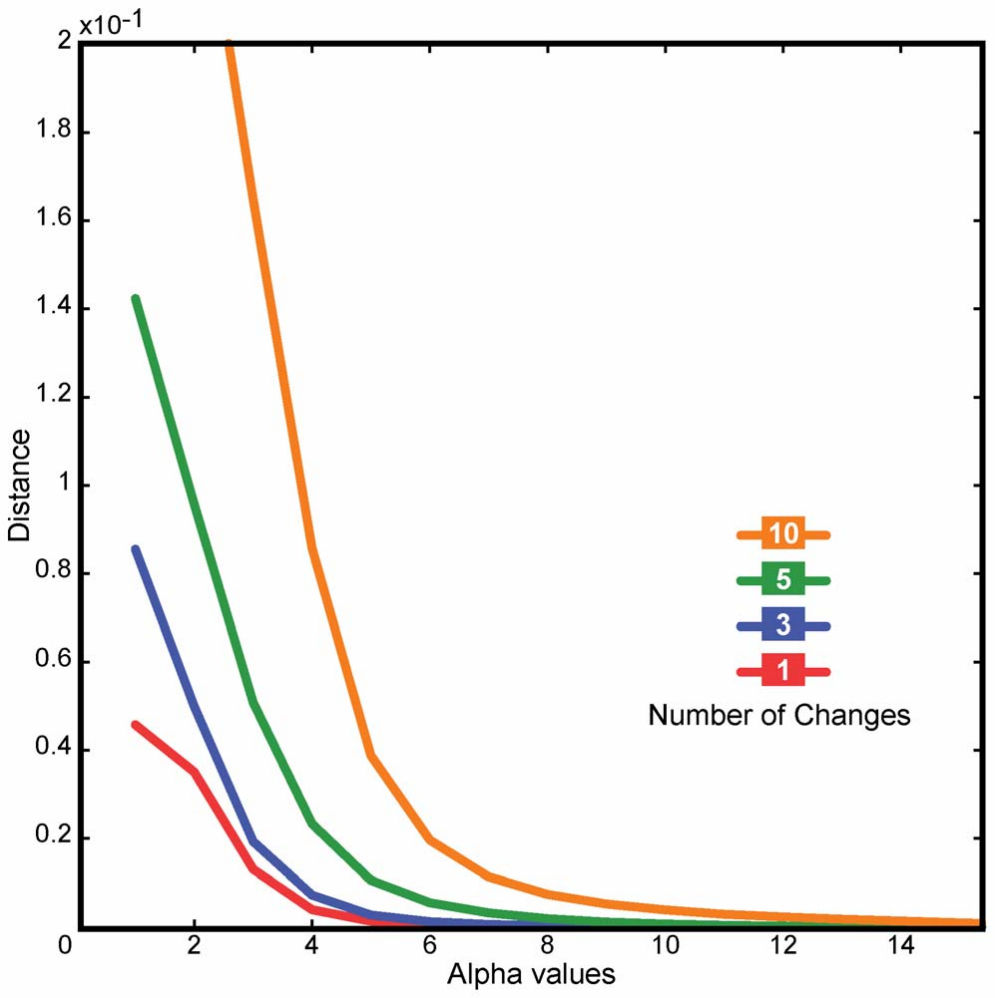
Similarity score for a sequence compared with modified versions of itself using different values of *α*. A 10,000 nt random sequence was created. Using this sequence as a template, a second was created that included one random substitution. The remaining sequences were built based on the last created sequence, adding new random substitutions. The result was an original sequence and four mutated sequences bearing 1, 3, 5, and 10 cumulative substitutions. The original sequence was then compared with each mutated sequence using different *α* values.

Our DNA sequence-to-signal mapping tool requires that different values be set for every possible doublet (i.e., 16 different values). For all the experiments presented in this section, we employed the values listed in Table 1. The proposed DNA sequence-to-signal mapping was designed to consider the nucleotides within a window defined by *α*. An example of the effect of *α* on the proposed mapping is depicted in Figure 1. As the value of *α* increases, the resulting DNA signal becomes smoother as the values corresponding to nucleotides within the window are combined. This indicates that the value of *α* determines how far a change is propagated through the signal. Note that a single nucleotide substitution produces a vertical shifting effect depending on the value of *α* with respect to a signal corresponding to a similar sequence. As *α* increases, a substitution has less impact on the signal shift. Indels in the DNA sequence are reflected as a horizontal shift with respect to another similar sequence proportional to the number of deleted or inserted bases. Figure 2 depicts the distance as computed by GAFD with respect to different numbers of changes in a given sequence employing different values of *α*. Note that, compared to methods that perform DNA sequence-to-signal mapping using individual nucleotides, *α* determines the robustness of our method with respect to subtle differences between the sequences being evaluated. In this work, we chose to employ 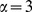 since this value allows us to distinguish between different numbers of signal changes.

**Table 1:** Values employed for DNA sequence-to-signal mapping.

Doublet	Value	Doublet	Value
AA	0	GA	8
AT	1	CA	9
TA	2	GT	10
AG	3	GG	11
TT	4	CT	12
TG	5	GC	13
AC	6	CG	14
TC	7	CC	15

In a sense, substitution matrices may be considered equivalent to simple–DNA-mapping functions. Where the former assigns a value to the difference between two nucleotides on different DNA sequences, the latter replaces the nucleotide with a number. Therefore, when comparing two mapped sequences, the difference between two nucleotides would be represented by their corresponding numbers. It becomes evident then that simple-mapping functions reduce the degrees of freedom when element-wise comparisons are made. The main advantage when mapping DNA is the ability to treat it as a series, which allows for the use of DSP and other concepts such as context information-dependent entities.

In this paper, we gathered contextual information using the NN algorithm, where each nucleotide value is considered along with its neighboring nucleotides. This approach, albeit still unidimensional, increases the degrees of freedom with respect to simple–DNA-mappings while also including contextual information. Although this is only a “proof of concept” study, we hypothesize that the referring context may not be only local, but also distant. Moreover, it may contain the sequence information itself as well as data from different annotation levels according to related known ontology or metadata. Adding the contextual elements will improve analysis by encompassing the DNA grammar into the structural information.

### GSP distance computations using GAFD

To evaluate the performance of GAFD with respect to existing methods for computing sequence distances, we assessed the similarity of unrooted phylogenetic trees generated by the NJ method [51], [52] (equal variance and independence of evolutionary distance estimates) using distance matrices computed with GAFD and NW (nuc44 scoring matrix, gap penalty of 8, and use of Jukes-Cantor for the maximum likelihood estimate of the number of substitutions) of various DNA sequences belonging to different organisms. In addition, we computed phylogenetic trees employing the Phylip method using ordinary parsimony and without randomization, with a search for the best 100 trees. The Phylip method was fed with sequences aligned using ClustalW with gap open penalty  = 10, gap extension penalty  = 0.05, and no weight transition. The resulting tree typologies were compared using the previously described cluster overlapping score 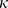.

#### Examination of the ribosomal S18 subunit gene

**Figure 3:**
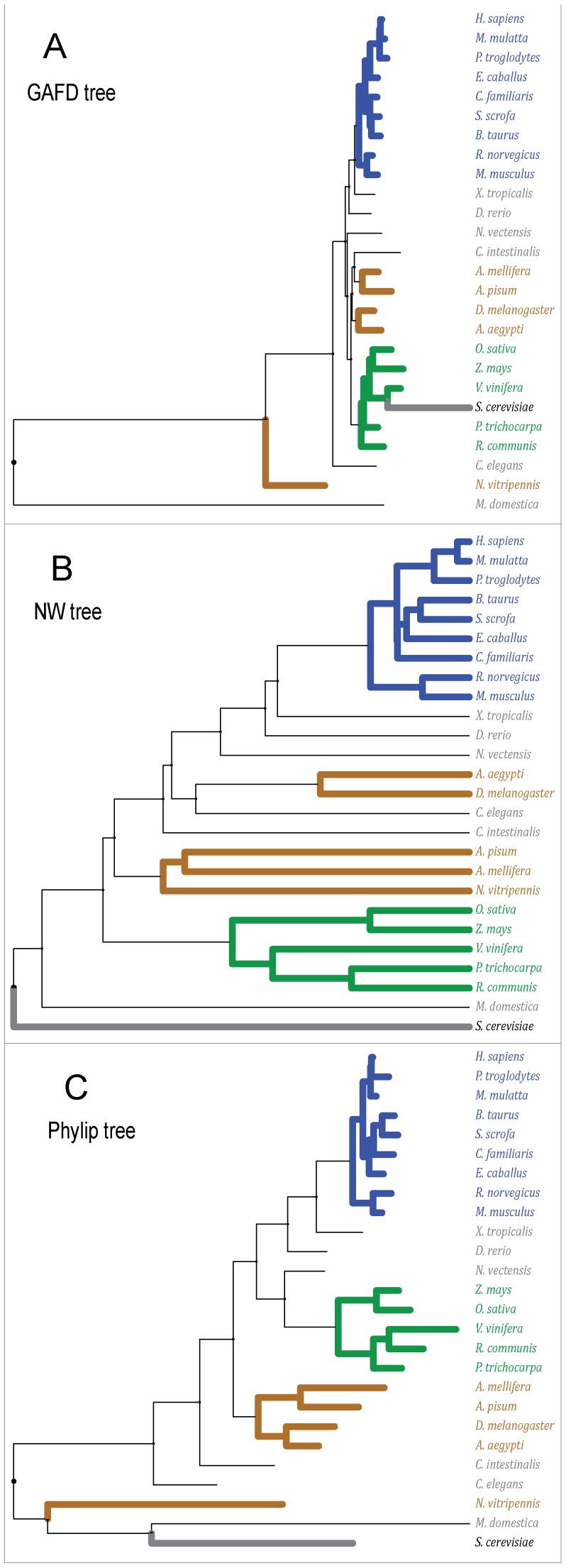
Depiction of phylogenetic trees for the ribosomal 18S subunit gene of 26 selected species. (A and B) Trees computed with GAFD and NW, respectively. (C) Maximum parsimony-bootstrapped Phylip tree. The species assessed and their corresponding KEGG entries are: *Acyrthosiphon pisum* (api:100145839), *Aedes aegypti* (aag:AaeL_AAEL009747), *Apis mellifera* (ame:552726), *Bos taurus* (bta:326602), *Caenorrhabditis elegans* (cel:Y57G11C.16), *Canis familiaris* (cfa:403685), *Ciona intestinalis* (cin:100182116), *Danio rerio* (dre:192300), *Drosophila melanogaster* (dme:Dmel_CG8900), *Equus caballus* (ecb:100052654), *Homo sapiens* (hsa:6222), *Macaca mulatta* (mcc:713939), *Monodelphis domestica* (mdo:100027117), *Mus musculus* (mmu:20084), *Nasonia vitripennis* (nvi:100117049), *Nematostella vectensis* (nve:NEMVE_v1g245261), *Oryza sativa* (osa:4334407), *Pan troglodytes* (ptr:455055), *Populus trichocarpa* (pop:POPTR_551159), *Rattus norvegicus* (rno:100360679), *Ricinus communis* (rcu:RCOM_0557270), *Saccharomyces cerevisiae* (sce:YDR450W), *Sus scrofa* (ssc:396980), *Vitis vinifera* (vvi:100245272), *Xenopus tropicalis* (xla:414719), *Zea mays* (zma:100285246). The trees are color-coded for the relevant phylogenetic groups, namely blue for eutherian mammals, green for plants and brown for insects. *S. cerevisiae* is bolded as reference.

Two experiments were performed by analyzing two sets of DNA sequences corresponding to the ribosomal S18 subunit (KEGG orthology K02964). This gene was selected because it is the broadest evolutionary marker discernable between all eukaryotes. In the first experiment, three basic clusters were built, namely mammals, insects, and plants, according to general taxonomy. The resulting phylogenetic trees generated from the distance matrices computed by the three methods are depicted in Figure 3. Note that the eutheryan (a mammal subgroup) were grouped in GAFD, NW, and Phylip. However, the insects were grouped differently by the three methods (e.g., *Nasonia vitripennis* was located far outside the other insects according to GAFD and Phylip). These results are consistent with the known complexity of insect genetics due to horizontal transference, spurious recombination, and high variability rate. Note that NW represented the outside eukaryote *Saccharomyces cerevisiae* appropriately, while GAFD placed it incorrectly among the plant group. Phylip placed this sequence in an outer group next to *Monodelphis domestica* and *N. vitripennis*. Although *M. domestica* was expected to be placed in an external group within mammals, it was placed in the outer branch of all trees. Lastly, with the exception of *S. cerevisiae* in GAFD, all plants were properly clustered.

In the second experiment, all entries of the aforementioned orthology were compared. A total of 149 organisms and 231 entries were analyzed, resulting in mean similarity scores of 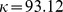 between GAFD and NW, 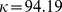 for GAFD and Phylip, and 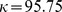 for NW and Phylip.

#### Assessment of other evolutionary markers

**Figure 4:**
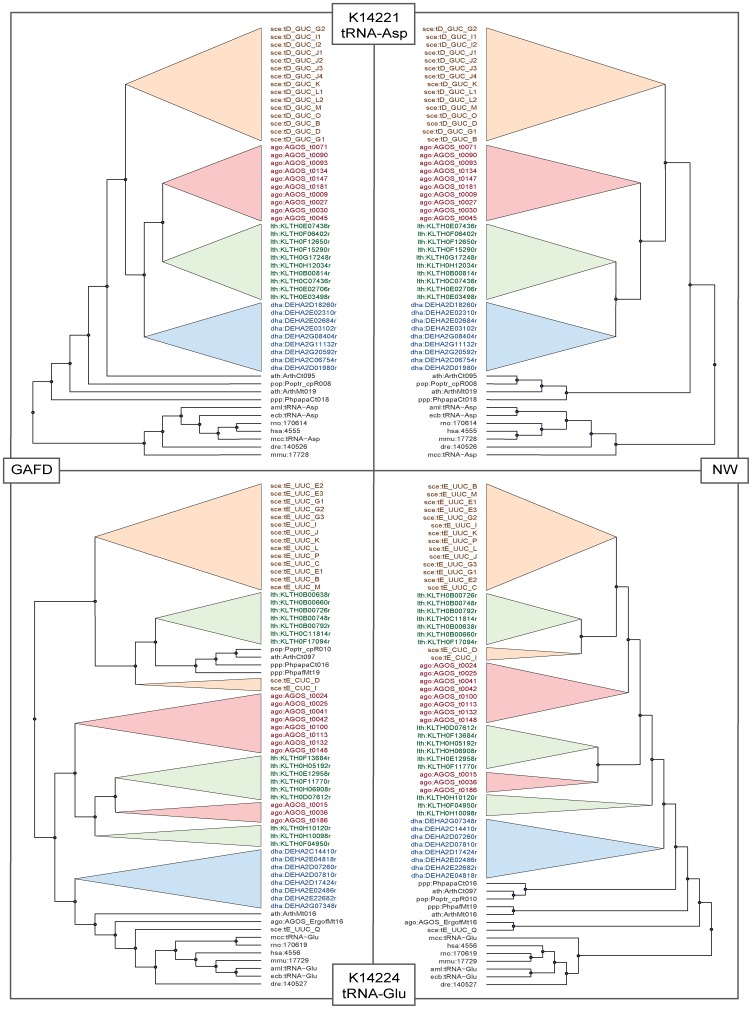
Phylogenetic trees generated by NW and GAFD for two selected orthologies: K14221 (tRNA-Asp) and K14224 (tRNA-GLU). The trees have been simplified to depict similarities in clustering. Each color represents a particular organism cluster: Orange: *S. cerevisiae*, red: *A. gossypii*, green: *L. thermotolerans*, blue: *D. hansenii*.

In this experiment, we selected evolutionary markers corresponding to coding (i.e., 21 tRNA synthetases and 2 ribosomal proteins) and non-coding (i.e., 20 tRNAs and 2 rRNAs) genes. We included species present in all KEGG orthologies and then selected all entries belonging to these organisms. We constructed and compared the phylogenetic trees generated using GAFD, NW, and Phylip. Figure 4 depicts two examples of trees generated by NW and GAFD for two selected orthologies (tRNA-Asp and tRNA-GLU). Note the similarity in gene clustering by GAFD and NW. Tables 2, 3, and 4 list the similarity scores 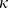 for the non-coding tRNAs, coding tRNA synthetases, and coding/non-coding ribosomal genes, respectively. The mean scores for the non-coding genes were 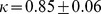, while 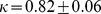 was exhibited for the coding genes. In general, the cluster overlapping scores between the methods were relatively high, indicating that GAFD can group similar sequences effectively.

**Table 2:** Similarity scores of tRNAs.

KEGG orthology	Gene name	NS	A	B	C
K14218	tRNA-Ala	68	85.13	80.86	95.21
K14219	tRNA-Arg	90	84.58	81.98	91.86
K14220	tRNA-Asn	46	86.18	80.83	89.33
K14221	tRNA-Asp	55	96.54	91.00	91.11
K14222	tRNA-Cys	24	91.27	86.26	93.00
K14223	tRNA-Gln	43	88.20	84.83	86.89
K14224	tRNA-Glu	65	88.22	89.53	92.00
K14225	tRNA-Gly	87	86.00	83.07	87.65
K14226	tRNA-His	33	79.80	79.97	93.64
K14227	tRNA-Ile	59	89.39	89.46	94.36
K14228	tRNA-Leu	91	88.85	90.53	91.74
K14229	tRNA-Lys	69	86.94	93.94	88.64
K14230	tRNA-Met	38	86.56	91.20	90.71
K14231	tRNA-Phe	44	85.80	86.03	89.64
K14232	tRNA-Pro	48	76.52	83.81	85.63
K14233	tRNA-Ser	84	89.88	90.99	95.30
K14234	tRNA-Thr	66	79.89	79.23	94.35
K14235	tRNA-Trp	33	92.94	90.08	88.09
K14236	tRNA-Tyr	41	88.55	86.82	92.96
K14237	tRNA-Val	74	83.73	85.95	95.69
	**Mean**		**86.75**	**86.32**	**91.39**
	**Std**		**4.48**	**4.28**	**2.92**

Cluster overlapping scores 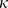 for the comparison of trees generated with (A) GAFD and NW, (B) GAFD and Phylip, and (C) NW and Phylip.

The species included in the comparisons are: *Ashbya gossypii, Ailuropoda melanoleuca, Arabidopsis thaliana, Debaryomyces hansenii, Danio rerio, Equus caballus, Homo sapiens, Macaca mulatta, Mus musculus, Lachancea thermotolerans, Populus trichocarpa, Physcomitrella patens, Rattus norvegicus, Saccharomyces cerevisiae.* NS = Number of sequences.

**Table 3:** Similarity scores for tRNA synthetase genes.

KEGG orthology	Gene name	NS	A	B	C
K01872	Alanyl-tRNA synthetase	24	85.11	80.07	84.71
K01887	Arginyl-tRNA synthetase	23	76.14	93.67	79.31
K01893	Asparaginyl-tRNA synthetase	32	89.44	91.00	85.96
K01876	Aspartyl-tRNA synthetase	29	87.88	91.10	86.28
K01883	Cysteinyl-tRNA synthetase	25	75.75	81.38	84.75
K01886	Glutaminyl-tRNA synthetase	16	76.87	80.29	85.32
K01885	Glutamyl-tRNA synthetase	22	93.44	89.99	90.27
K01880	Glycyl-tRNA synthetase	18	80.30	82.07	68.41
K01892	Histidyl-tRNA synthetase	23	82.39	80.93	93.00
K01870	Isoleucyl-tRNA synthetase	29	73.20	84.47	85.99
K01869	Leucyl-tRNA synthetase	28	84.43	80.77	78.68
K04567	Lysyl-tRNA synthetase, class II	24	72.61	83.08	75.03
K01874	Methionyl-tRNA synthetase	30	87.02	90.63	93.46
K01889	Phenylalanyl-tRNA synthetase	30	82.94	77.28	91.94
K01890	Phenylalanyl-tRNA synthetase	16	93.35	77.13	77.84
K01881	Prolyl-tRNA synthetase	23	84.01	87.98	79.11
K01875	Seryl-tRNA synthetase	30	74.98	73.18	90.28
K01868	Threonyl-tRNA synthetase	35	89.14	73.77	78.12
K01867	Tryptophanyl-tRNA synthetase	28	82.83	84.23	81.47
K01866	Tyrosyl-tRNA synthetase	30	79.04	73.57	87.78
K01873	Valyl-tRNA synthetase	25	78.60	86.56	78.30
	**Mean**		**82.36**	**83.01**	**83.62**
	**Std**		**6.10**	**6.01**	**6.36**

Cluster overlapping scores 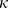 for the comparison of the phylogenetic trees generated with (A) GAFD and NW, (B) GAFD and Phylip, and (C) NW and Phylip.

NS = Number of sequences.

**Table 4:** Similarity scores for ribosomal protein genes and rRNAs.

KEGG orthology	Gene name	NS	A	B	C
K01982	Large subunit ribosomal RNA	65	75.33	93.57	81.04
K01979	Small subunit ribosomal RNA	64	76.99	87.50	81.13
K02963	Ribosomal protein S18	22	81.19	79.31	84.85
K02964	Ribosomal protein S18e	27	89.18	90.45	96.32
	**Mean**		**80.67**	**87.71**	**85.84**
	**Std**		**5.36**	**5.31**	**6.25**

Cluster overlapping scores 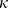 for the comparison of the phylogenetic trees generated with (A) GAFD and NW, (B) GAFD and Phylip, and (C) NW and Phylip.

NS = Number of sequences.

We assessed statistical significance by applying the non-parametric Wilcoxon Signed-Rank test, which does not require any assumptions regarding the normality of the data distribution. The null hypothesis for our tests is that the median difference 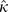 between the similarity of pairs of evaluations is not significant (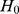: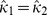), while the alternative hypothesis is the statistically significant difference between both medians (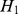: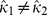). The resulting p-values for each test at a significance level of 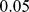 are listed in Table 5. No significant differences were observed between the three methods when examining coding sequences. However, for non-coding genes, GAFD performed differently from the other two methods. This may be related to the fact that coding genes appear to have a certain periodic structure [11], [16]–[23] which will affect GAFD since it also considers the frequency content of the mapped sequence.

**Table 5:** Statistical significance test results.

Type	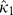	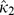	*H_0_*	p-value
NC	GAFD-NW	GAFD-Phy	Non rejected	4.56 ×10^−1^
NC	GAFD-NW	NW-Phy	Rejected	7.76 ×10^−3^
NC	GAFD-Phy	NW-Phy	Rejected	6.90 ×10^−4^
C	GAFD-NW	GAFD-Phy	Non rejected	3.70 ×10^−1^
C	GAFD-NW	NW-Phy	Non rejected	1.76 ×10^−1^
C	GAFD-Phy	NW-Phy	Non rejected	2.80 ×10^−1^

Wilcoxon Signed-Rank Test was performed to determine the statistical significance of comparing the means of the similarity scores 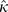 for each pair of methods on coding (C) and non-coding (NC) sequences.

Significance level of 0.05. Phy = Phylip.

**Figure 5:**
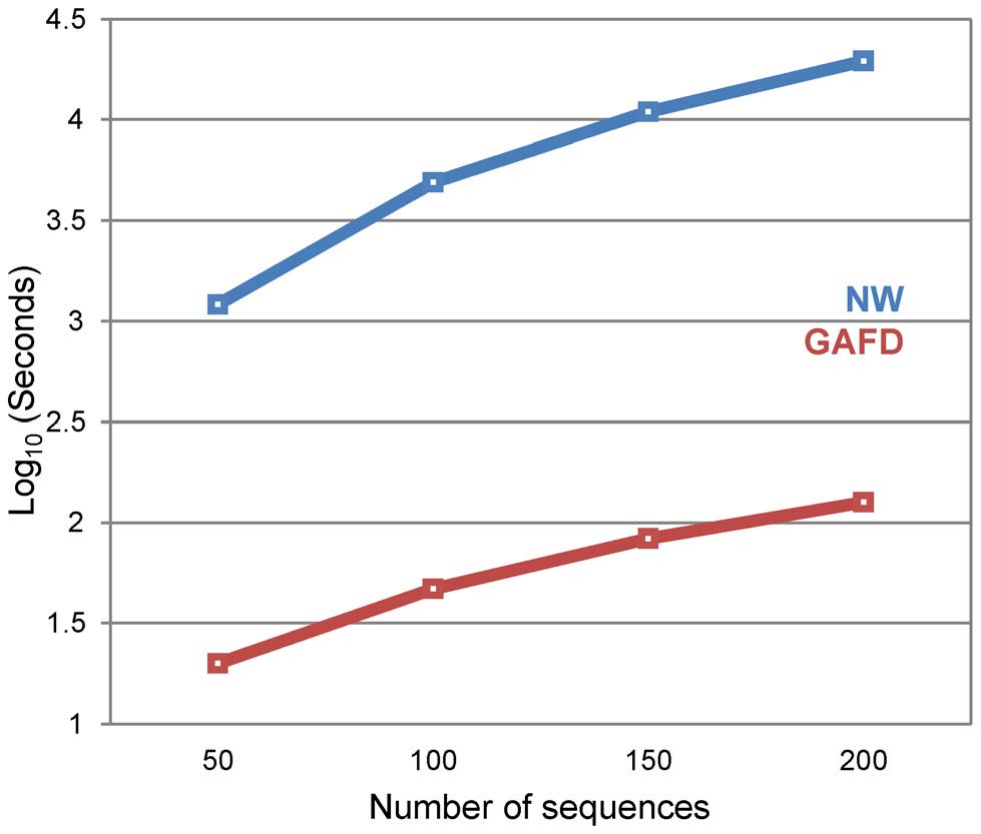
Times required to determine the distance matrix using NW and GAFD. A 10,000 nt random sequence was created. Using this sequence as template, another was created that included 10 random substitutions. The previously created sequence then became a template for the creation of a new sequence with 10 new random substitutions in non-mutated bases. The process was repeated until 20% of the sequence had changed. Then, both NW and GAFD were used to build distance matrices with an increasing number of sequences and the computer time was registered. Results are plotted on a logarithmic scale.

Figure 5 depicts the times required to compute the distance matrices using NW and GAFD on a desktop PC (i-Core 7, 2GHz, 6 GB RAM) for different numbers of sequences. GAFD performed faster than NW despite the implementation of high level MATLAB code. We believe that this performance could be improved by employing low level coding (e.g., C++) and tools such as GPU and parallel computing. A comparison of computer times for Phylip was not necessary because this method does not compute a similarity matrix.

Similar to other methods (e.g., [2]–[11]), the values presented in Table 1 are completely arbitrary. We expect that use of different mapping values will result in different distance scores. To evaluate the sensitivity of GAFD with respect to the dinucleotide values, we compared the phylogenetic trees corresponding to KEGG orthology K02964, generated using NW and Phylip, against 20,000 trees generated with GAFD using different random arbitrary dinucleotide values. The mean similarity scores were 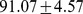 and 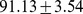 when compared to NW and Phylip, respectively.

Several elements may impact GAFD results to different extents. Even if they are related, these elements can be grouped according to the source of two critical phenomena, namely those related to the method, such as sequence-to-signal mapping and zero padding, and those related to the nature of the genomic grammar. Regarding the former, GAFD sequence-to-signal mapping is based on uniformly-euclidean unidimensional mappings. Even though comparisons will sometimes yield different results under particular sequence conditions, higher-order NN mappings are very difficult to implement and analyze. Additionally, since biologically meaningful sequences may not be of the same length, power spectra comparisons are influenced by zero padding. Regarding the latter, genomic information is typically full of challenging sequences, i.e. palindromes, inversions, translocations, repeats, duplications, and indels. All of these will exhibit distinct characteristics in power spectra that will in turn lead to inconsistencies when several sequence comparisons are performed. For example, differences among inversions, translocations, and palindromes may not be observed, while repetitions and duplications will display specific frequency peaks. How the interaction between all of these elements affects GAFD analysis is outside the scope of this paper. Moreover, NN mapping using context-sensitive information and DNA distance determination through power spectra comparison should be explored in the future.

GAFD is not intended for sequence alignment, but rather for comparing them in another domain and rendering a similarity value. We believe that, after refinement, this approach will enable us to discover relationships between sequences that are not bound to the sequence itself, but to specific underlying patterns in the genomic grammar that is yet to be fully understood.

### GSP distance descriptors

**Figure 6:**
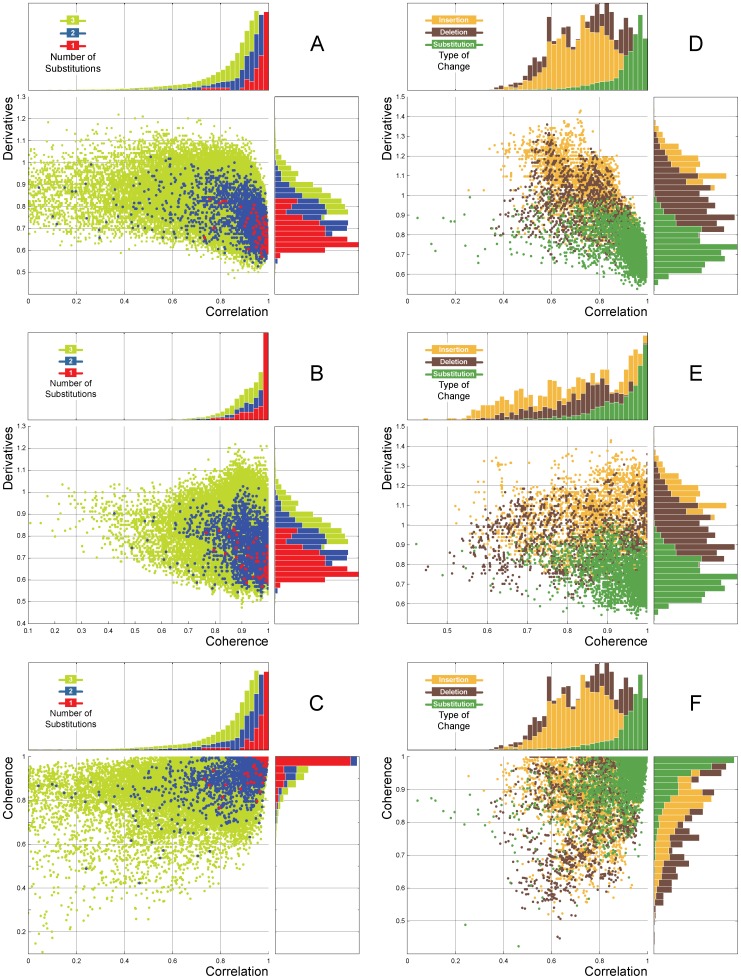
Depiction of the similarity space created with the three GSP distance descriptors. A random 20 nt sequence was created. Using this sequence as a template, all possible combinations for up to three substitutions were created and measured against the template using the three distance descriptors. The dots in A, B, and C correspond to the distances for one (red), two (blue), and three (green) substitutions, respectively. As expected, the more substitutions present, the farther they scattered along the frequency peak. Subsequently, starting with the same template, all possible combinations of insertions, deletions, and substitutions were created and measured similarly as aforementioned. The dots in D, E, and F correspond to the distances for insertions (yellow), deletions (brown), and substitutions (green). The distance scatters shift between substitutions and indels, which is especially evident in the Correlation and Derivative descriptors. The blue scatter on A through C is equal to the green scatter on D through F.

To explore the three-dimensional space generated by the proposed descriptors 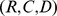, we performed an experiment in which we perturbed a randomly generated DNA sequence 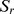 that generates a DNA signal 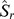 of length 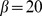. Using 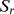 as the “mother sequence”, we generated all the DNA sequences and signals corresponding to all possible combinations of one, two, and three changes, considering all possible types of changes (i.e., substitutions, deletions, and insertions). Every pair of signals generated a point 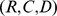 in the this space (Figure 6). Our results from the comparisons corresponding to one change were located near the origin, while those corresponding to two or three changes were positioned at increasing distance from the origin according to the number of changes. Additionally, the points corresponding to substitutions were well-separated from those corresponding to insertions and deletions (Figures 6 D and 6 E). These results demonstrate that GAFD can characterize the type of change present using a classification technique that combines several descriptors. However, coherence exhibited the poorest results since a lack of specificity for detecting insertions and substitutions was observed. This result is supported by Sims, et al. [53], where it was reported that optimal resolutions (length of 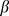) proved critical for genomic comparisons. Moreover, studies have shown that coherence AR models depend highly on the parameters employed [54].

**Figure 7:**
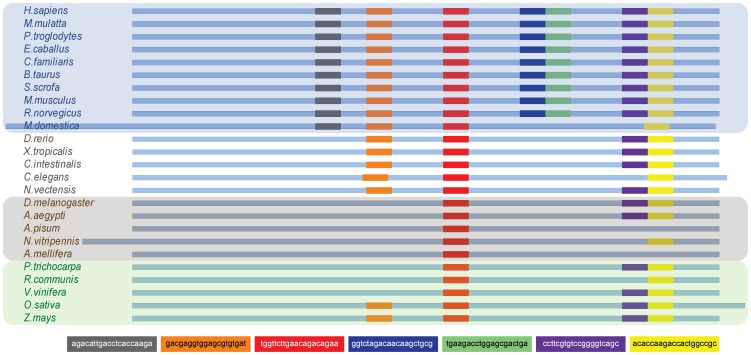
Schematic of the regions where selected entries of the signal dictionary were found in the different species.

As a preliminary domain search assay, we conducted another experiment using real data (i.e., ribosomal S18 subunit sequences from the previous 26 selected species). The signal corresponding to the *Homo sapiens* sequence was segmented into non-overlapping fragments of length 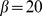 to generate a “signal dictionary”. From the dictionary, seven entries were selected at random and compared against the complete signal set employing a sliding window of length 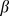. For each position within the sliding window, we computed the proposed similarity descriptors. We considered the segment of signal contained within the sliding window as similar to that from the dictionary if the correlation and coherence descriptors were larger than 0.9 and the comparison derivative was less than 0.8. The resulting alignment schematic is depicted in Figure 7. Even when the fragments were selected randomly, our results provide evidence that most mammals share similar fragments. Note that the number of shared fragments decreases as the sequences become less related to the original sequence (i.e., *H. sapiens*). Interestingly, insects shared the least number of fragments. These data suggest that it may be possible to determine biologically significant elements among compared sequences.

The use of alternative descriptors and classification techniques for grouping data is the subject of future work as it may be applied towards domain search and contig assembly.

## Conclusions

We present a novel GSP alignment-free method for determining the distance between two DNA sequences with a performance comparable to current methods such as Needleman-Wunsch and Phylip. Additionally, we evaluated three DSP-based distance metrics for use as descriptors for categorizing differences between pairs of DNA signal fragments. This work provides a foundation for the development of methods for domain search and the characterization of DNA sections.
